# A Case of Primary Signet-Ring Cell Cervical Carcinoma Treated with Chemoradiation, Brachytherapy, and Adjuvant Hysterectomy

**DOI:** 10.1155/2021/5544015

**Published:** 2021-12-27

**Authors:** Nicole Salmen, Dominic LaBella, Kenneth Strumpf, Wiley Douglas Bunn, Paul Aridgides

**Affiliations:** State University of New York Upstate Medical University, 750 E Adams Street, Syracuse, NY 13210, USA

## Abstract

Primary signet-ring cell carcinoma of the uterine cervix is a rare subtype of cervical mucinous adenocarcinoma. Approximately 20 cases of primary signet-ring cell carcinoma of the cervix have been reported. Pathologic examination shows that adenocarcinomas with mucin accumulation in intracytoplasmic vacuoles displacing the nucleus indicate signet-ring cell carcinoma. A thorough metastatic workup is needed both for staging and to rule out gastrointestinal tract origin. Due to the rarity of the disease, both the true incidence and optimal management are unknown. Herein, the authors present a case of stage 1B3 primary signet-ring cell cervical carcinoma treated with combined chemotherapy and radiation (including external beam radiation and brachytherapy), followed by resection for residual disease. This case is consistent with limited reports where all surviving patients received surgery as well as 1 surviving patient with bulky disease required with chemoradiation and adjuvant hysterectomy.

## 1. Introduction

Cervical cancer is the second most common cancer-related cause of death among women, causing 342,000 deaths annually worldwide [1]. Primary signet-ring cell carcinoma of the uterine cervix, one of the subtypes of cervical mucinous adenocarcinoma, is extremely rare [2]. A full metastatic workup is required to distinguish primary cervical signet-ring cell carcinoma of the cervix from metastatic disease from gastric, colorectal, or breast origin [2]. Stage I cervical carcinoma is successfully treated with either radical hysterectomy or radiation with or without concurrent chemotherapy, with primary chemoradiation favored for bulky disease [3]. Improved survival following adjuvant hysterectomy after chemoradiation has not been demonstrated in randomized trials [4] or meta-analyses [5]. Adjuvant hysterectomy is therefore generally discouraged following definitive chemoradiation and brachytherapy, however may be considered in select settings of persistent residual disease [6]. Herein, we report a case of bulky primary signet-ring cell cervical carcinoma where adjuvant hysterectomy was performed following definitive chemoradiation due to the unknown behavior of this extreme tumor and minimal but suspected residual tumor.

## 2. Case Presentation

A 50-year-old, gravida 2, para 2 perimenopausal woman presented with abnormal uterine bleeding requiring transfusion. The patient's past medical history is significant for anemia and psoriasis with an Eastern Cooperative Oncology Group performance status of 0. She had a recent negative pap smear 3 years prior and no history of abnormal pap results. Gynecologic consultation revealed a large, firm, bleeding, 6 cm mass in the cervix without clear evidence of parametrial or vaginal involvement. Biopsy under colposcopy confirmed high-grade invasive cervical adenocarcinoma. Transvaginal ultrasound showed a cervical mass with increased color Doppler flow and hypoechoic components. Computerized tomography of the abdomen and pelvis demonstrated a large heterogeneous mass at the cervix measuring 5 × 5 × 7 cm in size, with no obvious lymphadenopathy or distant disease.

**Figure 1:**
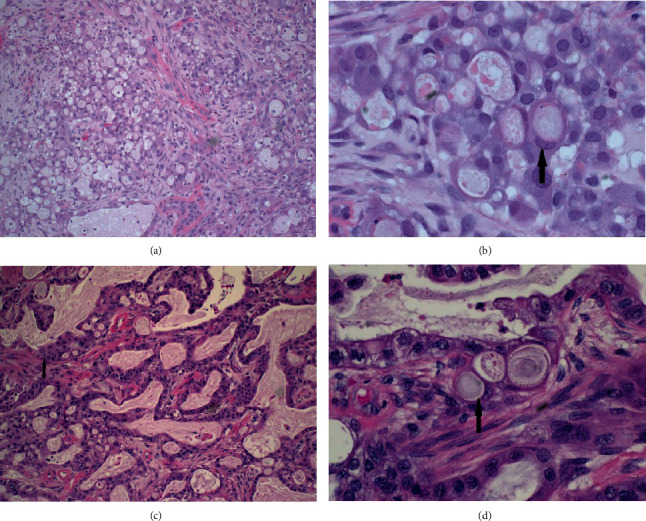
(a) Pretreatment biopsy containing sheets of malignant glandular cells. (b) Higher power magnification where the vast majority of tumor is composed of signet-ring adenocarcinoma cells (arrow). (c) Postsurgical biopsy demonstrating a mixture of conventional mucinous adenocarcinoma (arrow). (d) Postsurgical biopsy demonstrating residual signet cell adenocarcinoma (arrow).

The pretreatment biopsy (Figure 1(a)) contained sheets of malignant glandular cells. At higher power, the majority of tumor is composed of signet-ring adenocarcinoma cells (Figure 1(b), arrow), so named because the mucin-filled cytoplasm displaces the nucleus to the periphery of the cell giving the appearance of a signet ring. This is a highly unusual variant of cervical mucinous adenocarcinoma. An immunohistochemistry stain for P16, a marker for high-risk human papillomavirus, was strongly positive consistent with the diagnosis of cervical primary.

**Figure 2:**
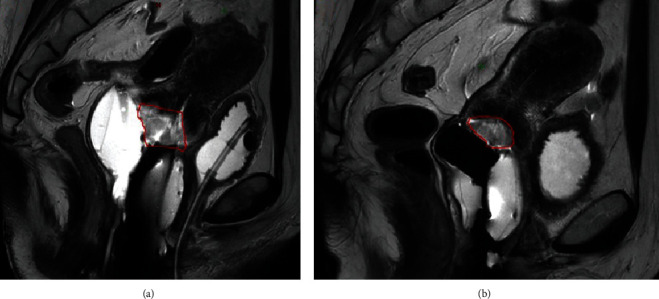
Magnetic resonance image sagittal scan with gross tumor volume (GTV) contours. (a) Fraction 1 with bulky tumor postexternal beam radiotherapy, where GTV was 20.5 cm^3^. (b) By fraction 4, there was a significant tumor response to brachytherapy with a GTV of 4.7 cm^3^.

At this point, it was recommended that the patient undergo external beam radiation therapy with chemosensitization followed by a brachytherapy boost. She underwent a course of concurrent chemoradiation with weekly cisplatin to the pelvis: 4500 cGy in 25 fractions followed by 5 fractions of high-dose brachytherapy. Brachytherapy consisted of 600-670 cGy per fraction to the high-risk clinical target volume (HRCTV) prescribed to cover 90% of the target (D90). The use of real-time MRI guidance on 2 fractions, and combined intracavitary/interstitial techniques, resulted in significant tumor shrinkage during brachytherapy that was evident between fractions 1 and 4 (Figure 2).

**Figure 3:**
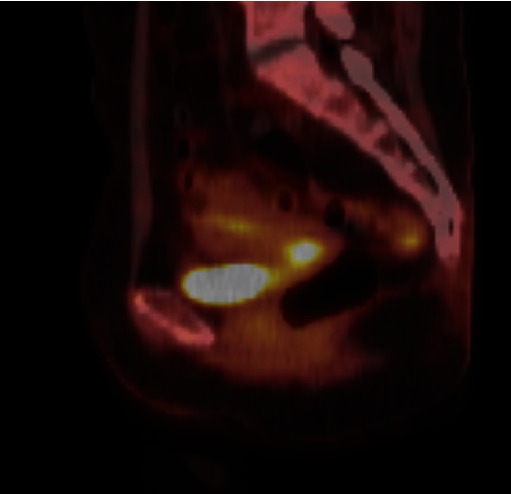
PET CT sagittal scan postradiation and presurgery with demonstration of a metabolically active mass within the cervix, consistent with the patient's known malignancy.

On pelvic exam at a one-month follow-up after brachytherapy completion, it was noted that the patient had a 1.5 cm, firm, nodular mass within the cervical canal suspicious for residual tumor. The cervix and uterus were freely mobile with no involvement of parametrial tissue, vaginal extension, anterior extension towards the bladder, or posterior extension towards the rectum. The tumor, cervix, and uterus were determined to be resectable with only minimal required resection of the paracervical tissue and vagina, and not necessitating a radical or exenterative approach [7]. A PET/CT revealed a standardized uptake value (SUV) of 13.97 in the cervix consistent with residual tumor, as seen in Figure 3. There remained no evidence of metabolically active lymph nodes or distal metastases.

After a multidisciplinary discussion of the institutional dedicated gynecology oncology tumor board which included specialists in gynecology oncology surgery, chemotherapy, and radiation, the options reviewed included adjuvant hysterectomy or continued close observation. The risks of adjuvant resection after chemoradiation were discussed and reviewed with the patient, including risk of poor wound healing and potential loss of function injury to bladder, rectum, and/or bowel. Close observation while having the possible benefit of not requiring further intervention and allowing further tumor regression was not favored by the tumor board or patient preference, given the high clinical suspicion of residual tumor that may ultimately require pelvic exenteration. The patient underwent adjuvant hysterectomy in the form of an exploratory laparotomy, total abdominal hysterectomy, and bilateral salpingo-oophorectomy. The hysterectomy specimen revealed residual cervical tumor measuring up to 1.6 cm in greatest dimension. Sections through this tumor demonstrated a mixture of conventional mucinous adenocarcinoma (Figure 1(c)) along with residual signet-cell adenocarcinoma (Figure 1(d), arrow). The relative predominance of the conventional mucinous adenocarcinoma in this specimen may represent a posttreatment alteration in the tumor.

At a 3-month postsurgery visit, the patient had no complaints, is without residual pelvic pain, and has maintained normal bowel and bladder function. Twelve months postsurgery, she continues to show no evidence of disease and the patient continues to feel well with no complaints.

## 3. Discussion

Primary signet-ring cell adenocarcinoma of the cervix is extremely rare, and it is more commonly found in the stomach or breast. To the best of our knowledge, approximately 20 cases of primary signet-ring cell carcinoma of the cervix have been reported, and due to the rarity of the disease, the true incidence is unknown. Giordano et al. found that a thorough metastatic workup is needed due to the low specificity of primary cervical diagnostic techniques [8]. Our case was p16 positive and had a negative metastatic workup, indicating a high-risk HPV etiology for primary signet-ring cell adenocarcinoma. HPV type 18 is a known risk factor for cervical adenocarcinoma, and almost all case reports of primary signet-ring cell cervical carcinoma are HPV type 18 positive [8, 9]. Pathologic examination shows that adenocarcinomas with mucin accumulation in intracytoplasmic vacuoles displacing the nucleus indicate signet-ring cell carcinoma [8].

Due to the stage 1B3 disease that was 7 cm in largest dimension, our patient was not a candidate for upfront surgery and received definitive chemoradiation with curative intent. While we typically reserve hysterectomy for salvage treatment, the rarity of her disease prompted PET/CT and multidisciplinary decision to perform adjuvant hysterectomy 6 weeks postchemoradiation. In a 2016 review of 18 reported cases of primary signet-ring cell cervical carcinoma, all 8 patients with stage IB disease were treated with surgery (1 of which following neoadjuvant chemotherapy) [9]. Seven of these 8 patients are reported to be surviving (0.5–10 years follow-up). In 2 of these 8 patients, adjuvant radiation was administered, and both are alive and disease free. Primary chemoradiation in cervical signet-ring cell carcinoma was only utilized in 1 patient with stage IIIB disease, who expired. This patient received chemoradiation and brachytherapy with a total point A dose of 75.2 Gy and unfortunately had a good clinical response initially but suffered local relapse within 1 year. A second case of chemoradiation for IIB primary signet-ring cell cervical carcinoma underwent hysterectomy as opposed to brachytherapy due to inadequate treatment response following 48.6 Gy [10]. This patient was disease free with 4 months of follow-up reported.

Our present case is the third reported case of chemoradiation with definitive intent for primary cervical signet-ring cell carcinoma. Similar to the outcomes of the prior 2 cases, we hypothesize she would have suffered local failure without adjuvant hysterectomy, given significant SUV uptake and active residual tumor on the pathology specimen. This is despite successful delivery of dose-escalated brachytherapy to high-risk CTV D90 (bioequivalent cumulative dose 97 Gy) using combined interstitial/intracavitary techniques, given bulky residual disease on fraction 1 (see Figure 2). A reasonable conclusion for bulky disease in primary signet-ring cell cervical carcinoma would be to anticipate incomplete or slow response to chemoradiation, and a lower threshold for requiring adjuvant hysterectomy may be necessary. This is limited to 3 reported patients exploring primary chemoradiation for cervical signet-ring cell carcinoma. Although our patient was 3 months follow-up postsurgery and is 7 months from diagnosis, this is a very rare primary tumor which may require unique considerations for treatment of bulky disease. Her surgery was successful with negative margins and no reported genitourinary or gastrointestinal morbidity. Although we typically would treat to a lower cumulative HR CTV dose if surgery was intended, her disease was not anticipated to become resectable per MRI and clinical evaluations prior to brachytherapy and therefore completed dose-escalated brachytherapy in 5 fractions.

In conclusion, our present case and review of the literature reporting outcomes for primary signet-ring cell carcinoma of the cervix suggest a role for surgery in both resectable and bulky disease. Results following primary radiation with or without chemotherapy have been discouraging. For cases that are unresectable, chemoradiation followed by adjuvant hysterectomy has been promising in 2 reported patients of which this is the second case.

## Data Availability

The patient clinical and imaging data used to support the findings of this study are restricted by the SUNY Upstate Institutional Review Board in order to protect patient privacy. Data are available from Paul Aridgides (https://aridgidp@upstate.edu) for researchers who meet the criteria for access to confidential data.

## References

[1] Sung H., Ferlay J., Siegel R. L. (2021). Global cancer statistics 2020: GLOBOCAN estimates of incidence and mortality worldwide for 36 cancers in 185 countries. *CA: a Cancer Journal for Clinicians*.

[2] Kawai S., Torii Y., Kukimoto I., Fujii T. (2019). A case of primary signet-ring cell carcinoma of the cervix containing full genome of human papillomavirus 16. *Indian Journal of Pathology & Microbiology*.

[3] Eifel P. J., Winter K., Morris M. (2004). Pelvic irradiation with concurrent chemotherapy versus pelvic and para-aortic irradiation for high-risk cervical cancer: an update of radiation therapy oncology group trial (RTOG) 90-01. *Journal of Clinical Oncology*.

[4] Keys H. M., Bundy B. N., Stehman F. B. (2003). Radiation therapy with and without extrafascial hysterectomy for bulky stage IB cervical carcinoma: a randomized trial of the Gynecologic Oncology Group. *Gynecologic Oncology*.

[5] Shim S. H., Kim S. N., Chae S. H., Kim J. E., Lee S. J. (2018). Impact of adjuvant hysterectomy on prognosis in patients with locally advanced cervical cancer treated with concurrent chemoradiotherapy: a meta-analysis. *Journal of Gynecologic Oncology*.

[6] Chino J., Annunziata C. M., Beriwal S. (2020). Radiation therapy for cervical cancer: executive summary of an ASTRO clinical practice guideline. *Practical Radiation Oncology*.

[7] Querleu D., Morrow C. P. (2008). Classification of radical hysterectomy. *Lancet Oncology*.

[8] Giordano G., Pizzi S., Berretta R., D'Adda T. (2012). A new case of primary signet-ring cell carcinoma of the cervix with prominent endometrial and myometrial involvement: immunohistochemical and molecular studies and review of the literature. *World Journal of Surgical Oncology*.

[9] Sal V., Kahramanoglu I., Turan H. (2016). Primary signet ring cell carcinoma of the cervix: a case report and review of the literature. *International Journal of Surgery Case Reports*.

[10] Kaidar-Person O., Amit A., Berniger A., Ben-Yosef R., Kuten A., Bortnyak-Abdah R. (2013). Primary signet-ring cell adenocarcinoma of the uterine cervix: case report and review of the literature. *European Journal of Gynaecological Oncology*.

